# MRI characteristics of chemotherapy-related central neurotoxicity: a pictorial review

**DOI:** 10.1186/s13244-023-01602-7

**Published:** 2024-01-17

**Authors:** Mina F. G. Isaac, Rugaiyah Alkhatib, Chi Long Ho

**Affiliations:** 1 Department of Radiology, Heartland Hospital, University Hospitals Birmingham NHS Foundation Trust, Birmingham, UK; 2 Department of Diagnostic Radiology, Sengkang General Hospital, Sengkang Eastway, Sengkang, 110 Singapore; 3 Duke-NUS Medical School, 8 College Road, Singapore, Singapore; 4 Yong Loo Lin School of Medicine, National University of Singapore, Singapore, Singapore

**Keywords:** Chemotherapy, Neurotoxicity, MRI, Brain, Patterns

## Abstract

The relentless advancement of chemotherapeutic agents has enhanced survival rates among cancer patients. However, this success comes with an increased prevalence of chemotherapy-induced neurotoxicity, which often mimics the symptoms of metastatic disease or paraneoplastic syndromes and poses a diagnostic challenge for clinicians. Imaging, particularly MRI, plays a pivotal role in unraveling this conundrum.

This comprehensive review explores the MRI patterns associated with central neurotoxicities induced by various chemotherapeutic agents. Our objective is to provide radiologists and clinicians with illustrative diagrams that offer a structured approach to diagnosing these conditions. By enhancing the understanding of these distinctive MRI patterns, we aim to facilitate accurate and timely diagnosis, ultimately improving patient care in the context of evolving cancer treatments.

**Critical relevance statement**

This article describes the essential role of MRI in identifying distinct patterns of chemotherapy-induced central neurotoxicity, enabling early diagnosis and improved patient care within the field of clinical radiology.

**Key points**

• Chemotherapy-induced neurotoxicity is a growing concern for cancer patients, and MRI is a key tool in diagnosis.

• This review highlights distinctive MRI patterns associated with various chemotherapy-induced neurotoxicities.

• Understanding these patterns improves patient care, ensuring timely intervention and accurate diagnosis in the complex world of cancer treatment.

**Graphical Abstract:**

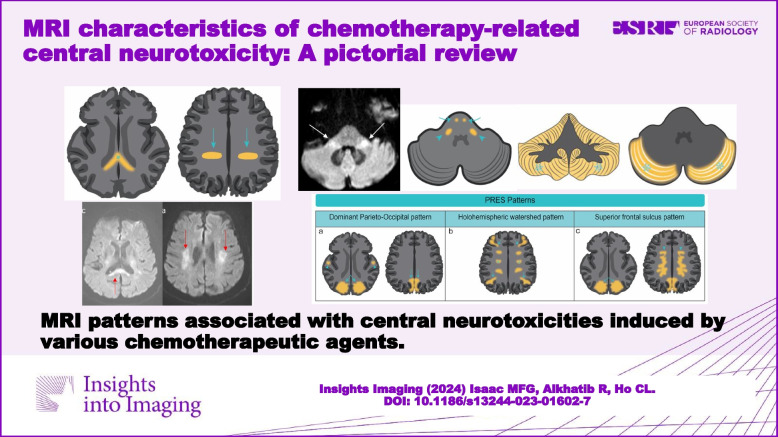

## Introduction

Chemotherapeutic agents play a critical role in cancer treatment, and they are classified based on their chemical structure and mechanism of action [1]. This classification includes various categories of drugs, such as alkylating agents, antibiotics, antimetabolites, topoisomerase inhibitors, microtubular targeting agents, platinum compounds, and newer classes like programmed death-1 (PD-1) or immune checkpoint inhibitors [1]. Every class of chemotherapeutic agent has its unique mode of action and spectrum of activity. These agents often have various side effects despite their efficacy in combating cancer. Among these, neurotoxicity is a particularly concerning issue affecting both the central nervous system (CNS) and the peripheral nervous system [2]. The extent and nature of neurotoxicity can vary based on factors such as the specific drug used, its dosage, and the duration of treatment.

The term “chemotherapy-induced neurotoxicities” encompasses a broad spectrum of conditions that affect the nervous system. These conditions include encephalopathy, posterior reversible encephalopathy syndrome (PRES), long-term cognitive impairment, acute pancerebellar syndrome, aseptic meningitis, oncologic therapy-related CNS infections, neurovascular complications, isolated neurologic symptoms, myelopathy, and radiculopathy [2]. Each of these conditions presents a unique set of challenges for both patients and healthcare providers.

This review focuses on the patterns observed in magnetic resonance imaging (MRI) associated with chemotherapy-induced central neurotoxicity. Understanding these patterns and their correlation with specific chemotherapeutic agents can aid in the early recognition, management, and mitigation of neurotoxic side effects in cancer patients. By shedding light on the radiological aspects of chemotherapy-induced neurotoxicity, we aim to contribute to the broader understanding of the neurological complications that can arise during cancer treatment.

## Methodology

In order to gather information on chemotherapy-induced central neurotoxicity and the associated MRI patterns, we conducted a comprehensive literature search. Our search strategy aimed to identify relevant studies and articles that focused on this topic. The following steps were taken:

### Literature search

We conducted an electronic literature search using the PubMed database. Our search strategy combined relevant keywords and synonyms to cover a range of terms associated with chemotherapy and neurotoxic conditions. Specifically, we included synonyms for “chemotherapy” or the names of specific chemotherapeutic agents, combined with terms related to neurotoxicity and specific neurotoxic conditions such as encephalopathy, cerebellar syndrome, and PRES.

### Language restrictions

We limited our search to articles published in English, as our analysis was focused on English-language literature.

### Screening

The studies retrieved from the initial search underwent an initial screening. During this stage, we assessed the relevance of each study based on its title and abstract. Studies that were clearly unrelated to our topic were excluded.

### Full-txt assessment

After the initial screening, we selected studies that appeared to be relevant based on the title and abstract. These selected studies underwent a thorough full-text assessment to ensure their suitability for inclusion in our review.

### Reference list review

In addition to the electronic search, we reviewed the reference lists of the included studies to identify any supplementary articles that might have been missed during the initial search.

This systematic approach allowed us to identify and include relevant articles and studies that provide insights into chemotherapy-induced central neurotoxicity and the associated MRI patterns. By compiling and analyzing this information, we aimed to create a comprehensive review of the topic.

## Review

MRI is a critical tool for the assessment of chemotherapy-induced central neurotoxicity. While the MRI findings often manifest as non-specific white matter changes or toxic leukoencephalopathy, it is important to note that certain chemotherapeutic agents may produce distinct and recognizable patterns in imaging. This review aims to provide an illustrative overview of such patterns in the context of chemotherapy-induced neurotoxicity. It is worth emphasizing that this article focuses on MRI patterns associated with drug-induced neurotoxicity and does not delve into other concurrent issues, such as CNS infections that may occur during chemotherapy treatment.

### MRI patterns of chemotherapy-induced central neurotoxicity

#### Acute-subacute leukoencephalopathy with reversible DWI pattern

Chemotherapy-induced encephalopathy is the most common central neurotoxicity [2]. While DWI abnormalities typically indicate irreversible cytotoxic brain injury, certain chemotherapeutic agents, such as methotrexate (MTX), 5-FU, and capecitabine, may produce reversible DWI lesions (Fig. 4).

**Fig. 1:**
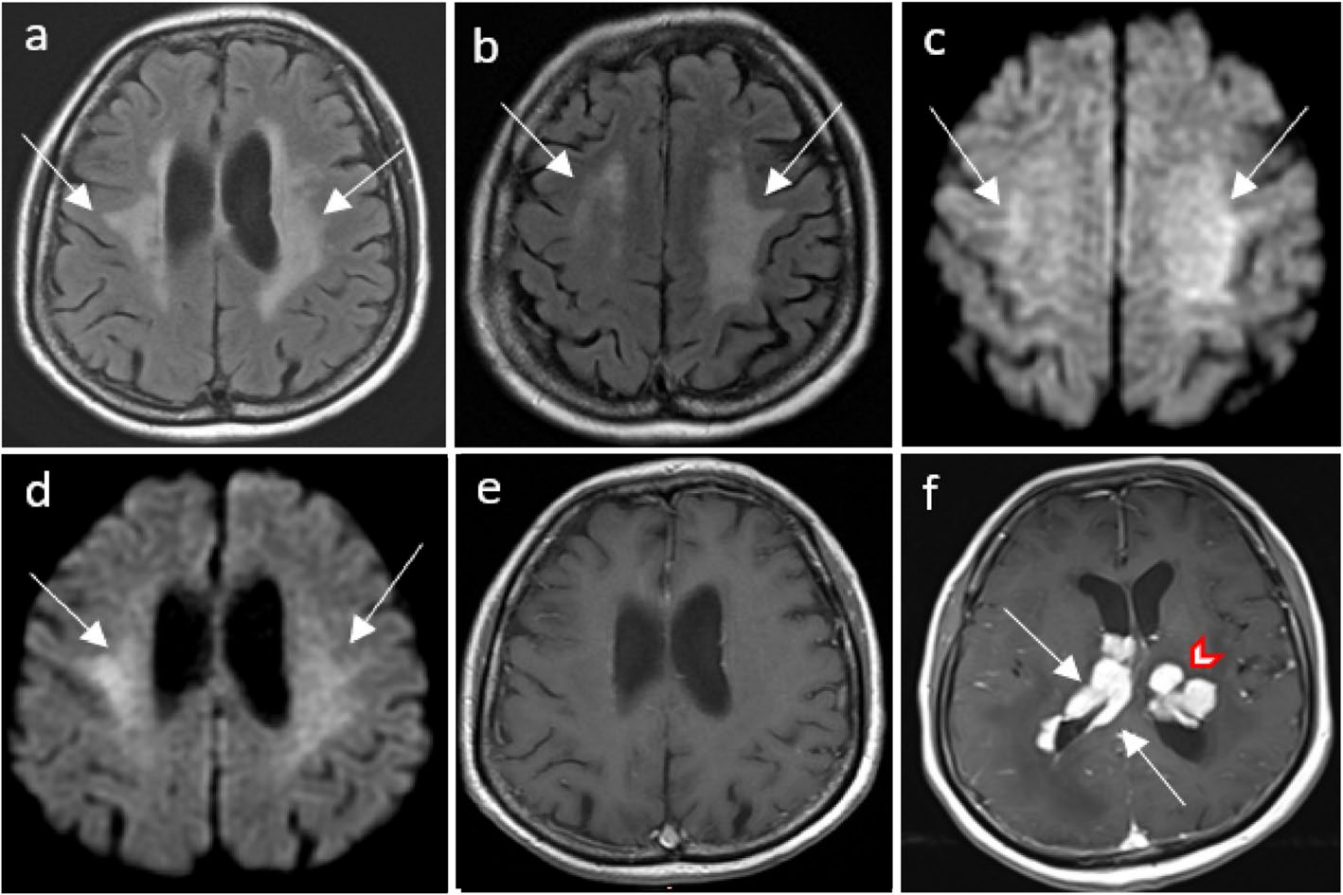
Methotrexate induced encephalopathy. A 64-year-old immunocompetent female has been diagnosed with primary central nervous system (CNS) lymphoma. High dose of methotrexate-based chemotherapy was administered. In the second week of treatment, she complained of bilateral upper limb weakness and numbness, as well as dysphagia. Axial FLAIR images (**a**, **b**) show hyperintensities in bilateral centrum semiovale and corona radiata sparing the subcortical U-fibers. Axial diffusion-weighted images (DWI) (**c**, **d**) show corresponding increased signal and low apparent diffusion coefficient (ADC) (not shown). The aforementioned areas with FLAIR and diffusion abnormalities do not show any post-contrast enhancement, and they represent methotrexate-induced leukoencephalopathy. Axial contrast-enhanced T1-weighted images (**e**, **f**) show enhancing lesions in both lateral ventricles including the ependymal and subependymal regions (arrows, **f**) as well as the left thalamus (red outlined arrowhead, **f**) are consistent with lymphomatous lesions

MTX neurotoxicity (Fig. 1) manifests as focal areas of restricted diffusion with T2/FLAIR hyperintensities in the deep white matter. Involvement can be unilateral or bilateral, either symmetrical or asymmetrical [3], occasionally affecting the splenium of the corpus callosum, known as the reversible splenial lesion (RSL) [4].

**Fig. 2:**
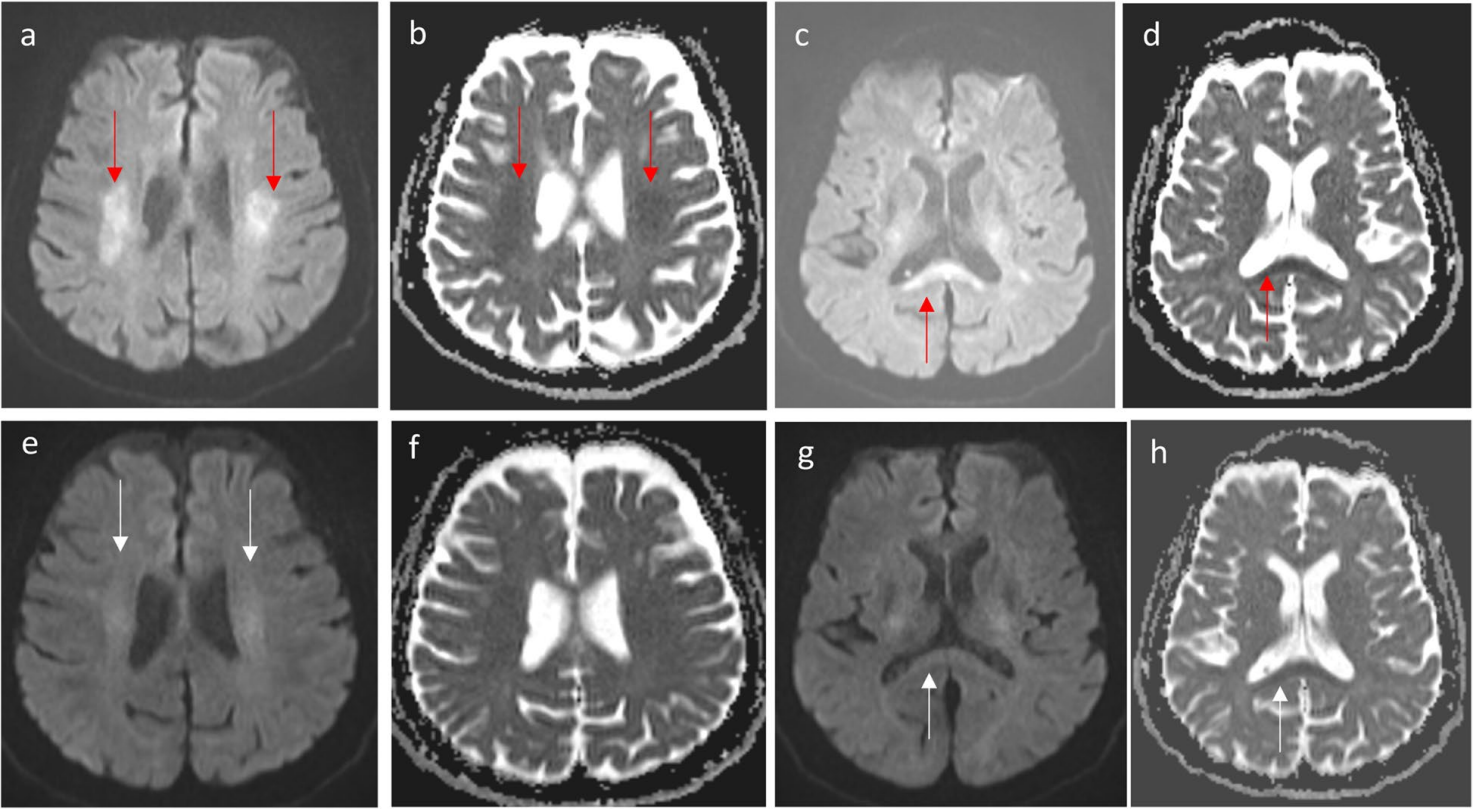
Capecitabine-induced leukoencephalopathy*.* An elderly patient on capecitabine for colon adenocarcinoma presented with transient expressive dysphasia and unsteady gait. Axial diffusion-weighted imaging (DWI) (**a**, **c**) and axial apparent diffusion coefficient (ADC) (**b**, **d**) MRI demonstrates restricted diffusion in the corona radiata (arrows in **a**, **b**) and splenium of the corpus callosum (arrows in **c** and **d**). These findings are compatible with capecitabine-induced leukoencephalopathy. A follow-up MRI after cessation of capecitabine administration showed near-complete resolution of the diffusion changes in bilateral corona radiata (arrows in **e**) and splenium of the corpus callosum (arrows in **g** and **h**) on the axial DWI (**e**, **g**) and ADC (**f**, **h**) images

**Fig. 3:**
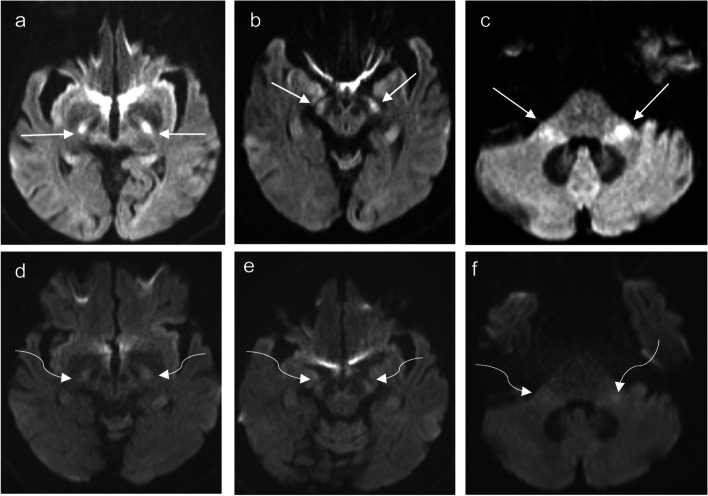
Capecitabine-induced leukoencephalopathy. An elderly patient on capecitabine for colon adenocarcinoma. Axial diffusion-weighted imaging (DWI) (**a**–**c**) MRI demonstrates a high signal in the posterior limb of bilateral internal capsules (arrow, **a**), bilateral cerebral (arrow, **b**), and bilateral middle cerebellar peduncles (arrow, **d**). The aforementioned areas of high DWI signal are associated with low ADC signal (not shown) consistent with restricted diffusion. Follow-up MRI after cessation of capecitabine administration showed near-complete resolution of the diffusion changes in the posterior limb of internal capsules (curve arrow, **d**), bilateral cerebral (curve arrow, **e**), and middle cerebellar peduncles (curve arrow, **f**)

**Fig. 4:**
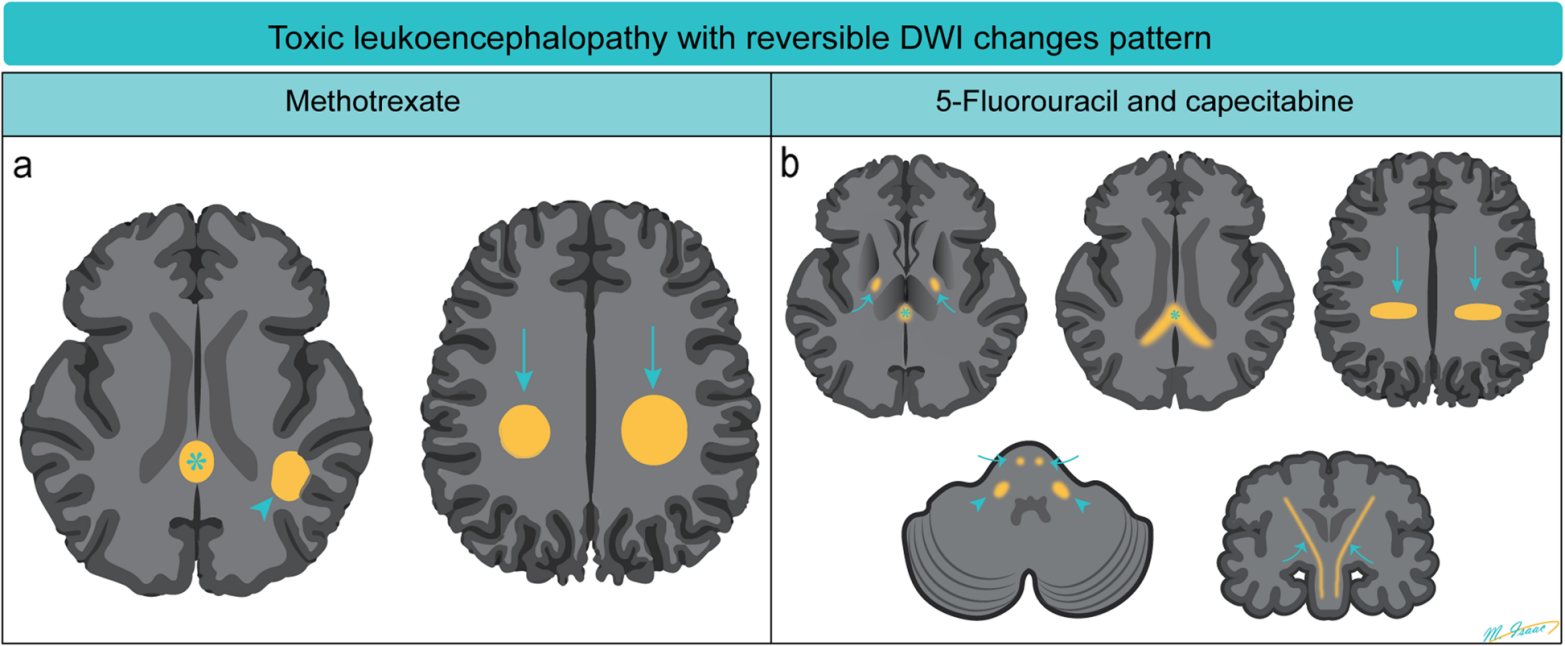
Diagrammatic illustration of MRI diffusion-weighted imaging (DWI) changes in Methotrexate (MTX) and 5-FU/capecitabine central neurotoxicity. **a** In MTX-related neurotoxicity, DWI changes typically involve the centrum semiovale (arrows) and corona radiata (arrowhead) and may be unilateral, bilateral, symmetrical, or asymmetrical in location. Rarely, reversible splenial lesion (RSL) may occur (star). **b** In 5-FU and capecitabine neurotoxicity, DWI changes involve the corona radiata (arrows), deep white matter, corticospinal tracts (curved arrows), middle cerebellar peduncles (arrow heads), and the splenium of the corpus callosum (RSL) (stars). Rarely, lesions may involve bilateral basal ganglia and thalami

Neurotoxicity associated with 5-FU and capecitabine (Figs. 2 and 3) is characterized by symmetrical periventricular DWI changes observed in the corona radiata, deep white matter, corticospinal tracts, middle cerebellar peduncles, and similarly to MTX neurotoxicity, the splenium of the corpus callosum (RSL) [5]. Similar MRI changes have been reported in bilateral basal ganglia and thalami [6]. The presence of reversible DWI patterns is a unique feature in chemotherapy-induced leukoencephalopathy. In routine MRI practice, hyperintensities on DWI often indicate irreversible cytotoxic brain injury, underscoring the diagnostic significance of these reversible patterns. Recognizing these reversible changes can aid in the early identification and management of central neurotoxicity related to chemotherapy, potentially allowing for timely intervention and improving patient outcomes (Fig. 4).

#### Acute cerebellar syndrome and reversible acute cerebellar toxicity (REACT) patterns

**Fig. 5:**
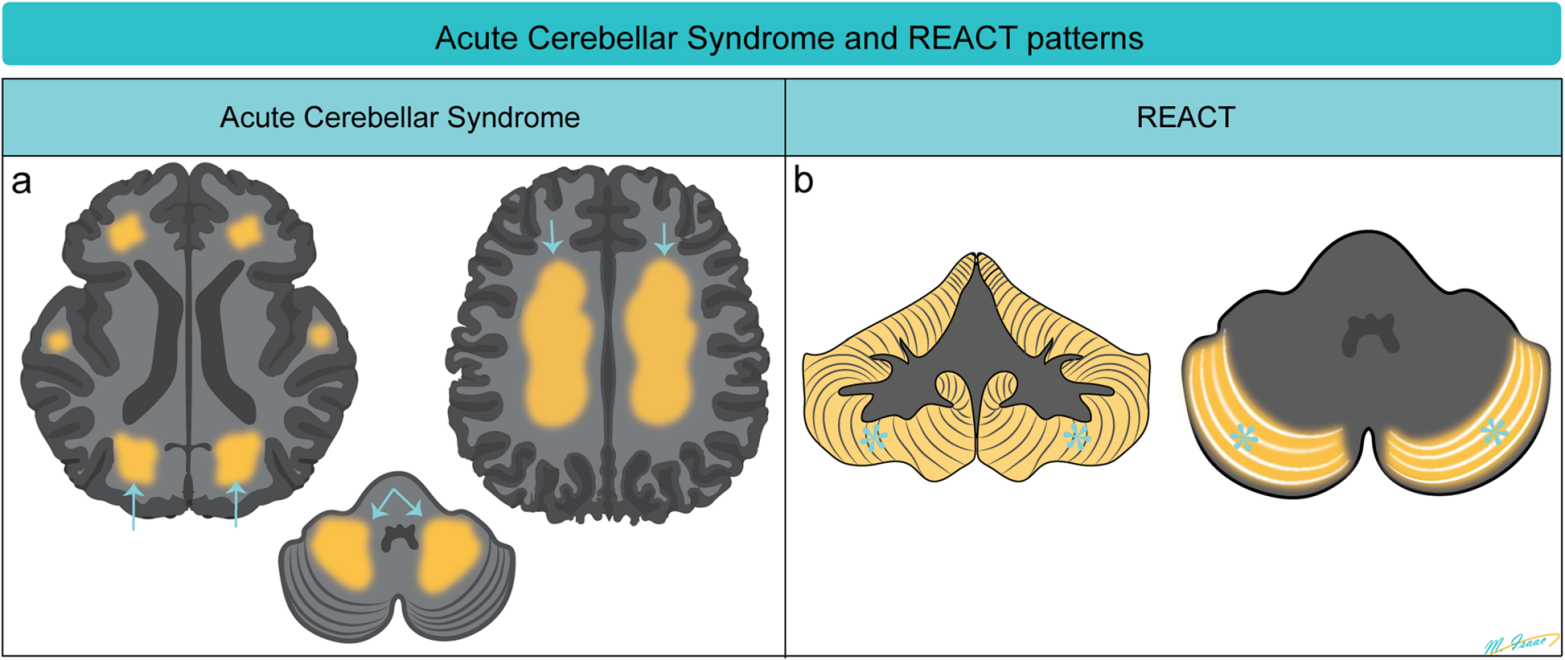
Diagrammatic illustration of MRI changes in acute cerebellar syndrome (**a**) and reversible acute cerebellar toxicity (REACT) syndrome (**b**). In acute cerebellar syndrome, MRI may show reversible diffuse white matter supra- and infratentorial T2/FLAIR hyperintensities (**a**, arrows). In REACT syndrome, the typical MRI characteristics are restricted diffusion involving the cerebellar cortices (**b**, stars) sparing the cerebellar deep nuclei and white matter as well as the supratentorial compartment

Acute cerebellar syndrome is a clinical condition characterized by dizziness, ataxia, dysarthria, vertigo, nausea, vomiting, and eye movement disorders. The syndrome is often associated with high doses of cytarabine, followed by 5-FU and capecitabine. It is rarely associated with other chemotherapy drugs, including MTX, vincristine, bortezomib, rituximab, and trastuzumab [7]. MRI findings in acute cerebellar syndrome may vary. The MRI may appear normal in some cases, while reversible white matter T2/FLAIR hyperintensities are observed in both supra- and infra-tentorial regions, reflecting acute toxic encephalopathy (Fig. 5).

REACT syndrome is a rare and potentially reversible encephalopathic syndrome associated with specific chemotherapy drugs including high-dose cytarabine [8] and Minnelide [9] (an experimental treatment for pancreatic and gastrointestinal cancers). It can present with symptoms such as gait ataxia, dysmetria, imbalance, speech impairment, and acute confusion [9]. MRI findings in REACT typically show restricted diffusion in the cerebellar cortex with a notable feature being the sparing of the deep cerebellar nuclei and supratentorial structures (Fig. 5) [9]. This distinctive pattern on MRI can help differentiate REACT syndrome from other neurological conditions. Recognizing these reversible MRI patterns is essential for diagnosing and understanding the central neurotoxic effects of the mentioned chemotherapy drugs.

#### Posterior reversible encephalopathy syndrome (PRES), also known as reversible leukoencephalopathy syndrome (PRLS)

**Fig. 6:**
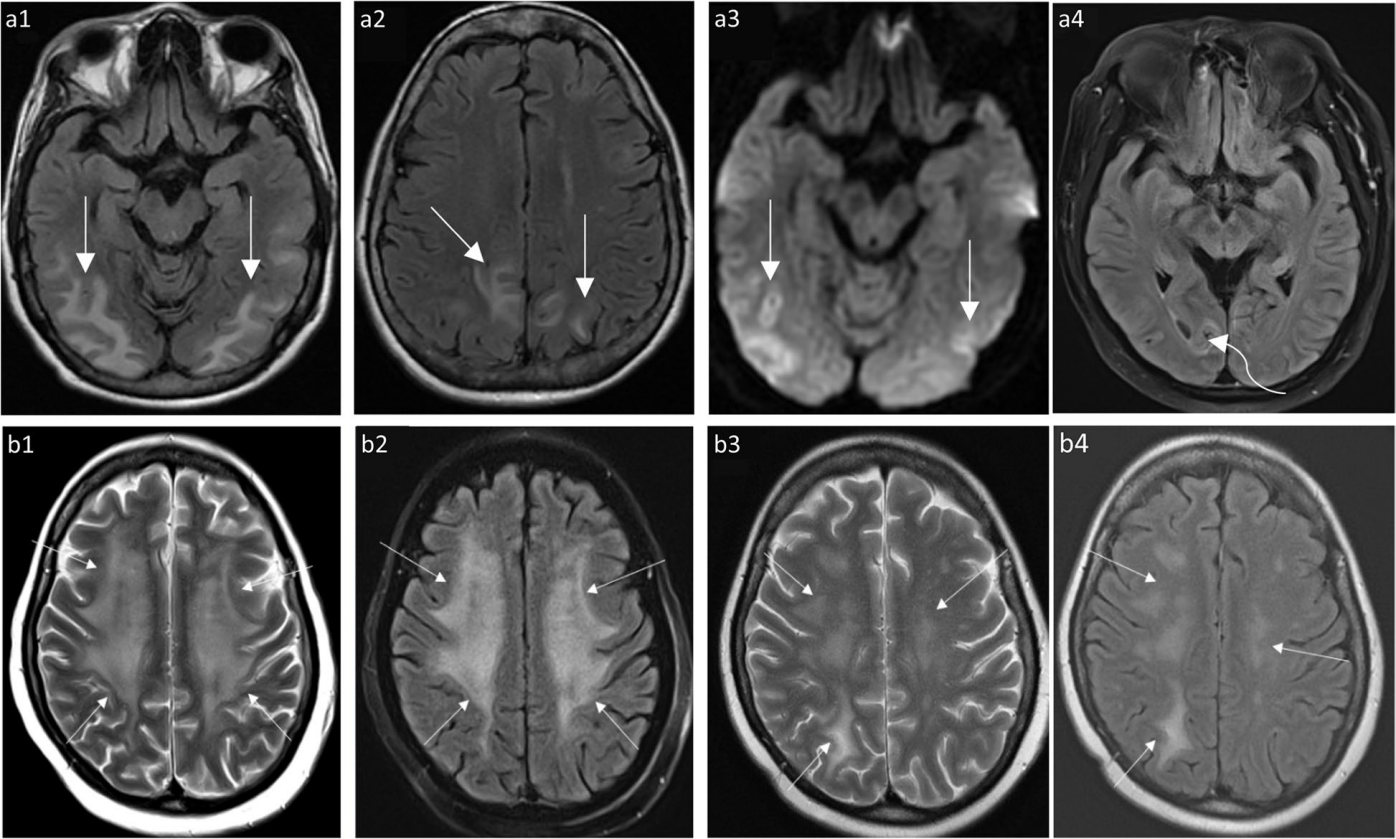
Examples of posterior reversible encephalopathic syndrome (PRES) related to chemotherapeutic agents. **a** A 52-year-old female with stage IV gallbladder carcinoma underwent a cisplatin-based chemotherapy regimen. Following the third cycle, she experienced worsening headache and giddiness, leading to hospital admission after two episodes of generalized tonic–clonic seizures. Axial FLAIR images (a1, a2) display hyperintensities in bilateral parietal, occipital, and posterior temporal lobes. The corresponding axial DWI image (a3) reveals increased signal and low apparent diffusion coefficient (ADC) (not shown), suggestive of PRES. A follow-up axial FLAIR image (a4) six weeks post-chemotherapy cessation shows near-complete resolution, with only residual signal in the right lingual gyrus (curved arrow). **b** A 45-year-old female with triple-negative breast cancer (TNBC) underwent mastectomy and received a Bevacizumab-based chemotherapy regimen. Axial T2 (b1) and FLAIR (b2) MRI brain images exhibit symmetrical hyperintensities in bilateral centrum semiovale and corona radiata (arrows), indicating Bevacizumab-related PRES. Subsequent MRI post-bevacizumab cessation demonstrates significant improvement in signal abnormalities on axial T2 (arrows, b3) and FLAIR (arrows, b4) images

PRES is an acute condition characterized by altered consciousness, visual disturbances, blindness, headaches, and seizures [10]. It is most commonly associated with hypertensive encephalopathy and conditions like preeclampsia or eclampsia. Several chemotherapeutic agents have been linked to the development of PRES, including cyclosporine and cyclophosphamide (an alkylating agent used in treatment of various malignancies). Less commonly, cisplatin (Fig. 6a), cytarabine, gemcitabine, ifosfamide, and vincristine have been associated with PRES [2]. Furthermore, certain immunomodulatory agents like bevacizumab (Fig. 6b), a monoclonal antibody inhibiting VEGFA (vascular endothelial growth factor 1), have also been reported to trigger PRES [10].

**Fig. 7:**
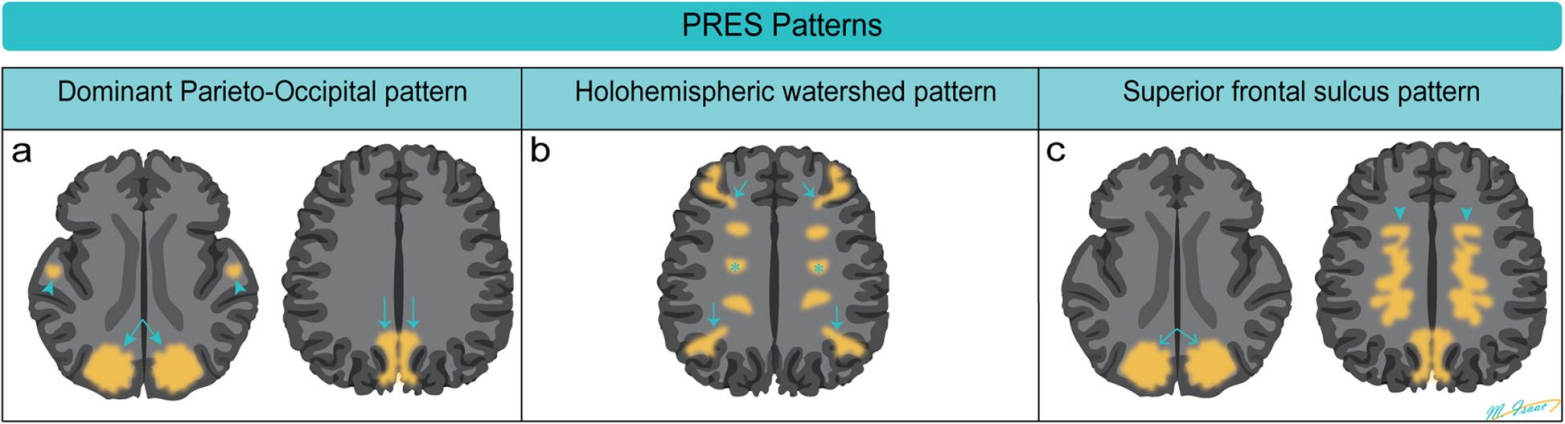
Diagrammatic illustration of three distinct imaging patterns of posterior reversible encephalopathic syndrome (PRES). **a** The classic occipitoparietal pattern is characterized by involvement of the parietal and occipital lobes (arrows) with variable involvement of the temporal lobes (arrowheads). **b** The holohemispheric watershed pattern is characterized by vasogenic edema in a linear pattern at watershed zones of the brain (arrows and stars). **c** The superior frontal sulcus pattern is characterized by varying degrees of involvement of the parietal and occipital lobes (arrows), associated with more isolated vasogenic edema of the mid to posterior aspect of the superior frontal gyri (arrow heads), sparing the frontal poles

The different imaging patterns of PRES, as classified by Bartynski, are summarized in (Fig. 7) [11]. Fortunately, PRES is typically reversible with symptomatic treatment and the discontinuation of the responsible chemotherapeutic agent [10].

Atypical PRES may involve other locations such as the brainstem, basal ganglia, thalami, and the deep white matter including the splenium of the corpus callosum. While spinal involvement is exceptionally rare, there have been reported instances [12].

Overall, PRES is a condition that can be triggered by various factors, including certain chemotherapeutic agents. Recognizing the distinct imaging patterns associated with PRES is crucial for timely diagnosis and effective management, leading to a high likelihood of reversibility and positive outcomes.

#### Chemotherapy-related neurovascular complications

Neurovascular complications associated with chemotherapy are relatively rare but can have significant consequences. Here is an overview of these complications:

Arterial ischemia and intracranial hemorrhage: Certain chemotherapeutic agents, including platinum-based agents like cisplatin, are associated with an increased risk of thromboembolic stroke. Additionally, agents such as 5-fluorouracil, gemcitabine, and bleomycin have been linked to a risk of arterial ischemia and intracranial hemorrhage [2, 7]. In the treatment of acute lymphoblastic leukemia (ALL), the use of L-asparaginase has been known to lead to cerebral venous thrombosis and intracranial hemorrhage [2]. Subdural hemorrhage may occur in high-dose regimens due to associated thrombocytopenia [2].

Reversible cerebral vasoconstriction syndrome (RCVS): RCVS is a clinical and radiological presentation characterized by acute thunderclap headache and reversible vasoconstriction of cerebral arteries [13]. It is considered a rare condition that can occur as a side effect of certain chemotherapeutic agents, particularly cyclophosphamide [14, 15]. The typical features of RCVS on magnetic resonance angiography (MRA) include diffuse multifocal cerebral vasoconstriction, sometimes resulting in a string-of-beads appearance and distal vascular pruning [16]. In some cases, approximately 17–38% of RCVS cases may exhibit PRES-like cerebral edema in the bilateral parieto-occipital regions, suggesting a possible coexistence of both conditions [16]. Other MRI features associated with RCVS include infarctions in watershed zones, small non-aneurysmal subarachnoid hemorrhages in cerebral sulci, and occasionally, intraparenchymal hemorrhages [16].

Neurovascular complications are a rare but serious concern associated with specific chemotherapy regimens. Early recognition and appropriate management are crucial for addressing these complications effectively. The imaging findings play a critical role in the diagnosis and management of these conditions.

#### Progressive multifocal leukoencephalopathy (PML) pattern

**Fig. 8:**
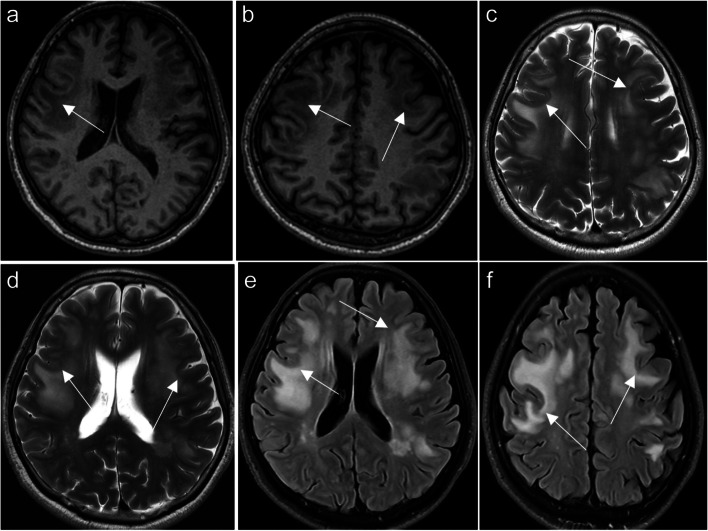
Fludarabine-induced progressive multifocal leukoencephalopathy (PML). A 56-year-old man with chronic lymphocytic leukemia had received a high dose of oral fludarabine 40 mg/m2/d for 5 days every 4 weeks for eight cycles earlier, developed progressive weakness of his left arm and legs. Axial T1-weighted (**a**, **b**), T2-weighted (**c**, **d**), and FLAIR (**e**, **f**). MRI revealed low T1 and high T2 and FLAIR signal intensities in bilateral cerebral white matter particularly involving the subcortical U fibers (arrows) giving a scalloped appearance. These findings are consistent with PML secondary to Fludarabine neurotoxicity

Progressive multifocal leukoencephalopathy (PML) is a rare and severe demyelinating disorder of the central nervous system (CNS) primarily caused by the JC virus. It typically occurs in immunocompromised individuals, but there have been reported cases related to chemotherapy agents [17]. Here is an overview of the PML pattern associated with chemotherapy:

Etiology: PML is almost exclusively found in immunocompromised patients, but in recent years, the incidence of medication-related PML cases has increased. Several chemotherapy drugs have been associated with PML, although such cases remain rare. Some of these drugs include rituximab, alemtuzumab, brentuximab, and fludarabine (Fig. 8) [18].

MRI: Typical MRI features of PML include the presence of unilateral or bilateral asymmetrical multifocal white matter lesions. These lesions have distinctive imaging characteristics, with low T1 and high T2 signal intensities. They typically involve the subcortical U or arcuate fibers, giving them a scalloped appearance [19]. As the disease progresses, these lesions become more confluent and may evolve into a round or oval shape, although they typically do not show post-contrast enhancement or mass effect [19, 20].

Location: PML lesions are more prevalent in the supratentorial brain, particularly in the frontal and parietal lobes [20]. Less frequently affected areas include the internal capsule, external capsule, and corpus callosum. The lesions can also involve the infratentorial compartment, including the middle cerebellar peduncle, pons, and cerebellum. Isolated lesions involving the cerebellar white matter and brainstem are rare, while spinal cord involvement is exceedingly rare [20].

Treatment: Medication-related PML is a rare but important consideration in the context of chemotherapy. Currently, the only effective treatment for PML is the withdrawal of the offending chemotherapy agents. This is done to help reestablish the patient’s immune system [7]. PML is a serious condition, and early recognition and intervention are vital.

#### Central neurotoxicity with immune checkpoint inhibitors (ICI)

Immune checkpoint inhibitors (ICI) have been successful in the treatment of various tumors, including melanoma [21], renal cell carcinoma [22], and non-small cell lung cancer (NSCLC) [23]. Pembrolizumab and Nivolumab, both Programmed death-1(PD-1) inhibitors, are among the ICI drugs approved for treatment of non-small cell lung carcinoma (NSCLC) by the Food and Drug Administration (FDA). However, ICI therapy is associated with a spectrum of immune-related adverse events (irAEs), including neurotoxicities [24]. CNS irAEs associated with ICI therapy include encephalopathy, ICI-induced autoimmune or limbic encephalitis, hypophysitis, PRES, multiple sclerosis (MS), aseptic meningitis, transverse myelitis, necrotizing myelopathy, and vasculitis [24, 25].

**Fig. 9:**
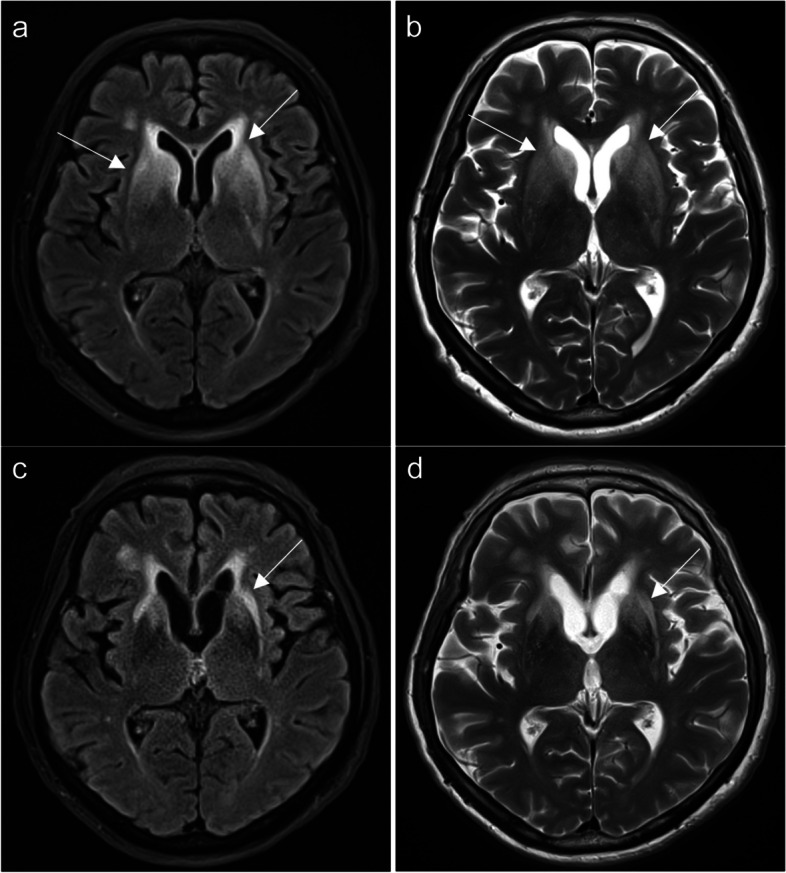
Pembrolizumab-induced toxic leukoencephalopathy. A 72-year-old female with non-small cell lung carcinoma (NSCLC) had received Pembrolizumab (200 mg intravenous administered every 3 weeks). Three days after the second cycle of Pembrolizumab, she was admitted with sudden onset confusion, ataxia, and cognitive impairment. Axial FLAIR (**a**) and T2-weighted (**b**) MRI brain scans reveal hyperintensities in bilateral periventricular white matter and bilateral lentiform nuclei, external capsules, and claustrum (arrows). These findings are consistent with Pembrolizumab-induced toxic leukoencephalopathy. She was given high-dose methylprednisolone administered intravenously at 1000 mg daily for 3 days, with a near-complete resolution of his expressive dysphasia and improvement in gait. She was discharged on a regimen of 60 mg of prednisolone daily, which was subsequently weaned by 10 mg each week. A Follow-up MRI brain one month after discharge reveals a significant reduction of the T2 and FLAIR hyperintensities in the basal ganglia as demonstrated on axial FLAIR (**c**) and T2-weighted (**d**) images

**Fig. 10:**
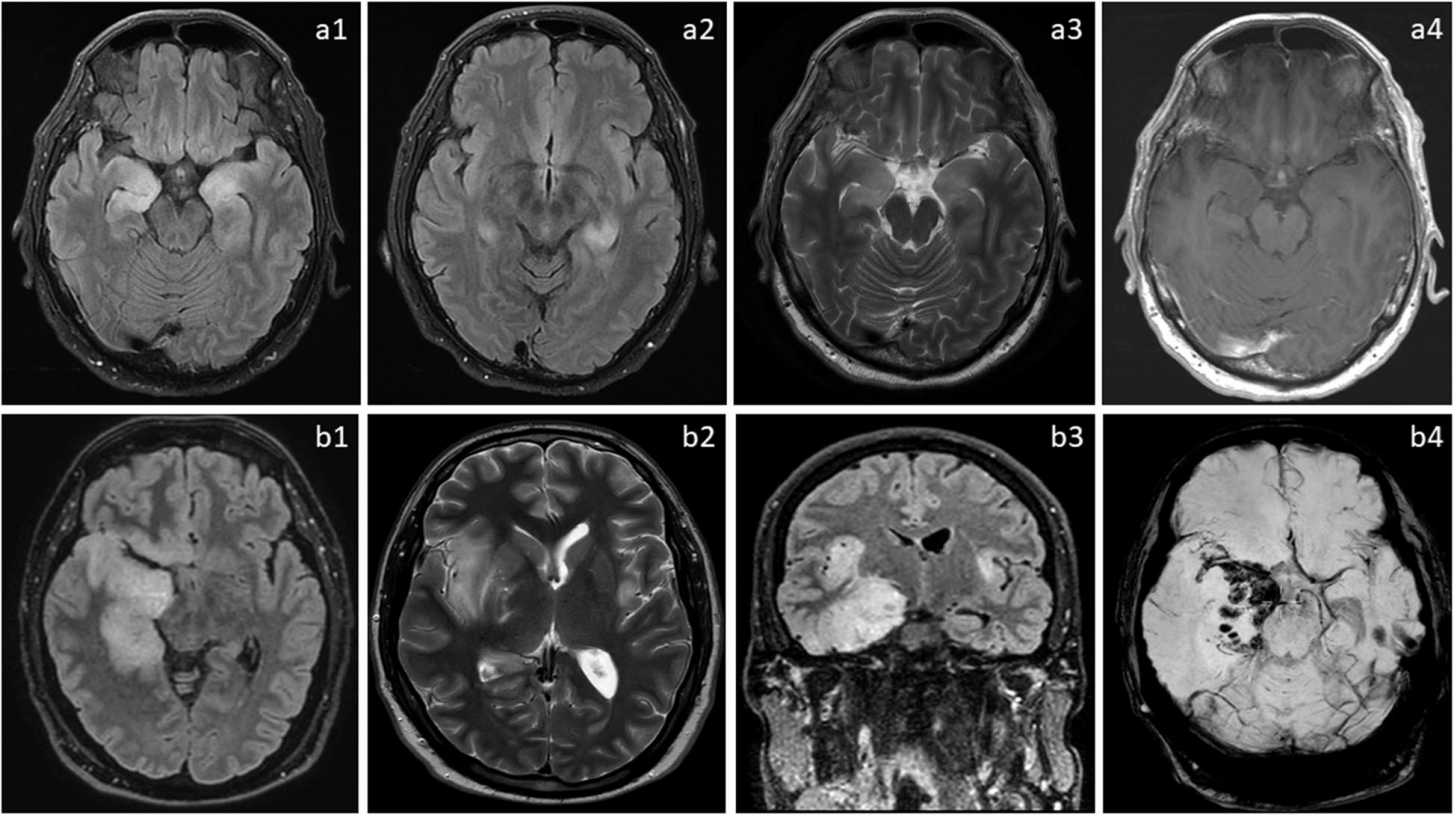
MRI characteristics of limbic encephalitis. **a** A 53-year-old male, presenting with headache and drowsiness, tested positive for Leucine-rich, glioma inactivated 1 (LGI1) antibody. MRI depicted symmetrical FLAIR (a1, a2) and T2 (a3) signal abnormalities in bilateral medial temporal lobes. No enhancement was observed in the T1 post-contrast sequence (a4). **b** Comparative case: highlighting a case of herpes encephalitis, a primary differential for autoimmune limbic encephalitis. A 36-year-old man diagnosed with herpes simplex encephalitis displayed involvement of the right medial temporal lobes and bilateral insular cortices, more severe on the right side, showcasing elevated FLAIR (axial b1 and coronal b3) and T2 (b2) signal intensities. Blooming artifacts in the right medial temporal lobe on SWI sequence (b4) suggested hemorrhage, favoring herpes simplex encephalitis over autoimmune encephalitis

In fact, Immune Checkpoint Inhibitor Associated Autoimmune Encephalitis (ICI-associated AE) is an uncommon complication, representing a relatively novel and not fully understood spectrum. Reported cases exhibit variability, with some individuals testing positive for autoantibodies while others do not [26]. ICI-associated AE lacks distinctive MRI patterns [27]; however, MRI may reveal T2/FLAIR hyperintensities affecting white and deep gray matter, including the lentiform nuclei and external capsule/claustrum, as illustrated in (Fig. 9). In addition, limbic encephalitis has also been reported with characteristic MRI appearance of symmetrical inflammatory changes in bilateral mesial temporal lobes (Fig. 10) [28]. The variability in MRI findings, coupled with often negative results in auto-immune antibody panels, contributes to diagnostic challenges. This emphasizes the importance of performing a thorough clinical and laboratory evaluation in suspected cases of ICI-associated AE [27].

MRI features in ICI-induced hypophysitis (ICI-H) include hypo-enhancing geographical areas of low T2 signal in the anterior pituitary without blooming artifact [29]. This pattern typically reflects fibrosis rather than necrosis or hemorrhage [29]. These specific MRI characteristics, providing a way to distinguish it from other types of hypophysitis or neoplasms. CNS irAEs are a notable consideration in patients receiving ICI therapy. Recognizing these neurotoxicities, their associated MRI patterns, and distinguishing features can be crucial for early diagnosis and appropriate management.

#### Spinal cord toxicity

**Fig. 11:**
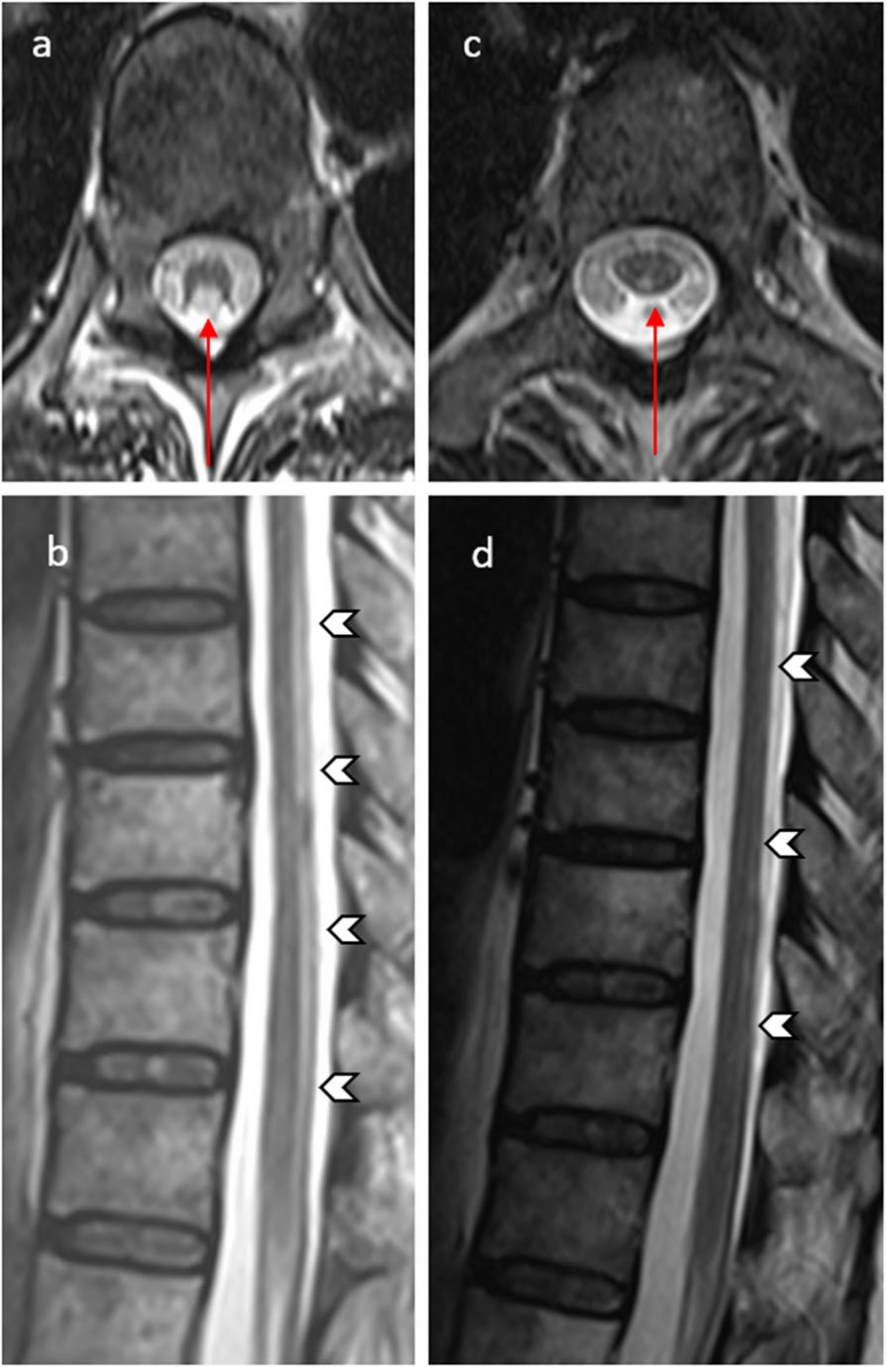
Methotrexate-induced myelopathy mimicking subacute combined degeneration of the spinal cord (SCID). A 54-year-old female with acute lymphocytic leukemia treated with intrathecal methotrexate (MTX) developed bilateral lower limb weakness and numbness approximately 2 weeks after commencing treatment. Axial (**a**) and sagittal (**b**) T2-weighted thoracic spine MRI demonstrates high signal lesions involving long segment along the dorsal column of the spinal cord (arrowheads, **b**). It resembles an “inverted V sign” or “kangaroo ears sign” on the axial image (red arrow, **a**) masquerading subacute combined degeneration of the cord. Axial (**c**) and sagittal (**d**) T2-weighted images at six-month follow-up MRI after cessation of intrathecal MTX treatment show improvement with significant reduction of the signal abnormality within the dorsal column of the spinal cord (red arrow in c and arrowheads in **d**)

Intrathecal MTX administration can sometimes lead to an irreversible condition that resembles sub-acute combined degeneration, even when vitamin B12 levels are normal [30]. This condition manifests as limb weakness, back pain, sensory changes, and urinary bladder dysfunction. MRI scans typically reveal T2 hyperintensities in the dorsal columns, giving a pattern that resembles an “inverted V,” often called the “kangaroo ears sign” (Fig. 11) [30].

**Fig. 12:**
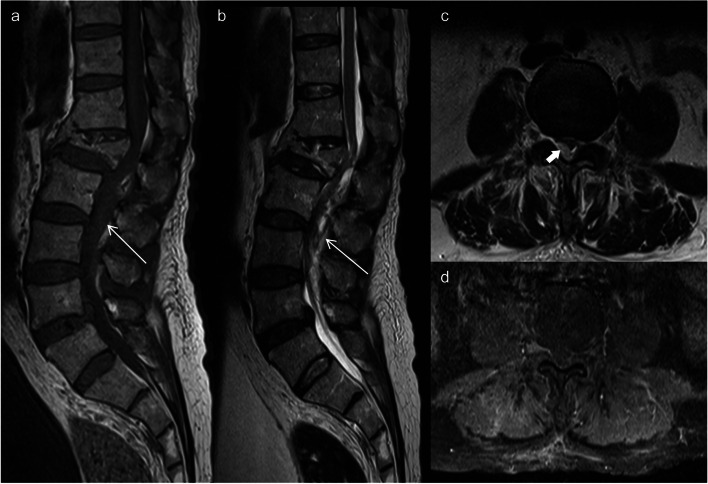
Intrathecal methotrexate (MTX) administration complicated by epidural hematoma. A 66-year-old man was on an intrathecal MTX regimen for treatment of non-Hodgkin lymphoma, presented with severe back pain that radiates to the left leg after the first week of intrathecal MTX administration. Sagittal T1- (**a**) and T2-weighted (**b**) MRI lumbar spine images demonstrate a posterior epidural collection of iso to slightly high T1 and mixed heterogeneous T2 signal intensities, compressing the thecal sac with crowding of cauda equina (arrows). Axial T2WI (**c**) shows corresponding high T2 intensity epidural collection compressing the right posterolateral aspect of the thecal sac (thick arrow). Axial T1W post-contrast image reveals no abnormal enhancement. The findings are consistent with an epidural hematoma. Besides, there is an old compression fracture of the L1 vertebral body which has been stable since the prior study (not shown). Patient underwent an urgent laminectomy and evacuation of the epidural hematoma with subsequent improvement of his symptoms

One of the complications that may arise from intrathecal MTX administration is lumbosacral polyradiculopathy, characterized by post-gadolinium enhancement of the anterior nerve roots of the cauda equina. Additionally, intrathecal chemotherapy may lead to myelopathy. Certain drugs, including cytarabine, cisplatin, carmustine, and thiotepa are reported to cause this condition [10]. Lastly, intraspinal or epidural hematoma has been reported as a rare complication following intrathecal administration of medications or chemotherapeutic agents (Fig. 12) [30].

These spinal cord toxicities can occur as a result of intrathecal chemotherapy, and their recognition through MRI findings is essential for early diagnosis and appropriate management. The kangaroo ears sign in subacute combined degeneration-like conditions is a distinctive MRI feature that can aid in diagnosis.

## Conclusion

In conclusion, this review has shed light on the various MRI patterns associated with chemotherapy-induced neurotoxicities. Cancer patients often present with a spectrum of neurological symptoms, making it challenging to differentiate between these neurotoxicities, tumor progression, and paraneoplastic syndromes. Understanding these distinctive MRI patterns is essential for accurate diagnosis and management within the complex landscape of cancer care.

The highlighted MRI patterns encompass a range of conditions, including reversible DWI changes, acute cerebellar syndrome, REACT, PRES, PRLS, PML, central neurotoxicity with immune checkpoint inhibitors, and spinal cord toxicity. These patterns, each with unique characteristics, provide clinicians and radiologists with valuable diagnostic tools.

The role of imaging findings in the diagnosis and monitoring of chemotherapy-induced neurotoxicities cannot be overstated. Early recognition of these neurotoxicities and their associated MRI patterns is critical for ensuring timely and appropriate management, ultimately improving the quality of care for cancer patients.

## Data Availability

Not applicable.

## Abbreviations

5-FU: 5-Fluorouracil
AE: Autoimmune encephalitis
CNS: Central nervous system
DWI: Diffusion-weighted imaging
FDA: Food and Drug Administration
ICI: Immune checkpoint inhibitors
irAEs: Immune-related adverse events
MRA: Magnetic resonance angiography
MRI: Magnetic resonance imaging
MS: Multiple sclerosis
MTX: Methotrexate
NSCLC: Non-small cell lung cancer
PD-1: Programmed death-1
PML: Progressive multifocal leukoencephalopathy
PRES: Posterior reversible encephalopathy syndrome
RCVS: Reversible cerebral vasoconstriction syndrome
REACT: Reversible acute cerebellar toxicity
RSL: Reversible splenial lesion
VEGFA: Vascular endothelial growth factor A
